# Determination of the Total Phenolics Content and Antioxidant Activity of Extracts from Parts of Plants from the Greek Island of Crete

**DOI:** 10.3390/plants12051092

**Published:** 2023-03-01

**Authors:** Eleftherios Kalpoutzakis, Theodoros Chatzimitakos, Vassilis Athanasiadis, Sofia Mitakou, Nektarios Aligiannis, Eleni Bozinou, Olga Gortzi, Leandros A. Skaltsounis, Stavros I. Lalas

**Affiliations:** 1 Department of Pharmacognosy and Natural Products Chemistry, University of Athens, 15771 Panepistimiopolis Zografou, Greece; 2 Department of Food Science and Nutrition, University of Thessaly, 43100 Karditsa, Greece; 3 Department of Agriculture Crop Production and Rural Environment, School of Agricultural Sciences, University of Thessaly, 38446 Volos, Greece

**Keywords:** antioxidant activity, plant extracts, Rancimat, food rancidity, free radical scavenging, differential scanning calorimetry

## Abstract

Oxidative damages are responsible for many adverse health effects and food deterioration. The use of antioxidant substances is well renowned, and as such, much emphasis is placed on their use. Since synthetic antioxidants exhibit potential adverse effects, plant-derived antioxidants are a preferable solution. Despite the myriads of plants that exist and the fact that numerous studies have been carried out so far, there are many species that have not been examined so far. Many plants under research exist in Greece. Trying to fill this research gap, the total phenolics content and antioxidant activity of seventy methanolic extracts from parts of Greek plants were evaluated. The total phenolics content was measured by the Folin–Ciocalteau assay. Their antioxidant capacity was calculated by the 2,2-Diphenyl-1-picrylhydrazyl (DPPH) scavenging test, the Rancimat method based on conductometric measurements, and the thermoanalytical method DSC (Differential Scanning Calorimetry). The tested samples were obtained from several parts of fifty-seven Greek plant species belonging to twenty-three different families. Both a remarkably high phenolic content (with gallic acid equivalents varying between 311.6 and 735.5 mg/g of extract) and radical scavenging activity (IC_50_ values ranged from 7.2 to 39.0 μg/mL) were found in the extract of the aerial parts of *Cistus* species (*C. creticus* subsp. *creticus*, *C. creticus* subsp. *eriocephalus*, *C. monspeliensis*, *C. parviflorus* and *C. salviifolius*), *Cytinus* taxa (*C. hypocistis* subsp. *hypocistis*, *C. hypocistis* subsp. *orientalis* and *C. ruber*), and *Sarcopoterium spinosum*. Furthermore, the sample of *Cytinus ruber* showed the highest protection factor (PF = 1.276) regarding the Rancimat method, which was similar to that of butylated hydroxytoluene (BHT) (PF = 1.320). The results indicated that these plants are rich in antioxidant compounds, potentiating their use either as food additives to enhance the antioxidant properties of food products, or protect them from oxidation, or as sources for the preparation of food supplements with antioxidant properties.

## 1. Introduction

Lipid peroxidation is a major cause of deterioration during processing and storage, which leads to losses of quality and nutritional value and the development of unpleasant flavors. In addition, oxidative stress, in which reactive oxygen molecules such as superoxide, hydroxyl, and peroxyl radicals are generated, has been suggested to be the cause of aging and various diseases in humans [1]. To overcome the abovementioned problems, the addition of antioxidants is required, since it assists in the preservation of flavor and color and in food quality deterioration avoidance. The most frequent antioxidants used to preserve food are the synthetic compounds butylated hydroxyanisole (BHA), butylated hydroxytoluene (BHT), propyl gallate, and tert-butyl hydroquinone. However, there are published reports regarding the disadvantages of synthetic antioxidants—for example, BHA or BHT—and their possible toxic properties for human and animal health [2,3]. On the other hand, epidemiological evidence indicates that the consumption of foodstuffs containing antioxidant compounds of plant origin (i.e., phytochemicals) is advantageous for human health [4]. So, nowadays, consumers assume that natural compounds are safer and, as such, prefer natural antioxidants to synthetic ones [3].

The majority of aromatic, spicy, medicinal and other plants contain chemical compounds that exhibit antioxidant properties. Therefore, their crude extracts are being used more and more in the food industry, resulting in an increased interest in related studies [5]. In addition, natural antioxidants have the potential to be used as constituents for the maintenance of health and protection from diseases, such as coronary heart disease and cancer. This fact has resulted in the rising interest among scientists and food manufacturers, as well as consumers, who move toward functional foods with specific health effects [6]. However, scientific information on the antioxidant properties of various plants, particularly those that are less widely used in cuisine and medicine, is still rather scarce.

So far, several researchers have screened a large number of herbs to evaluate their antioxidant activity. For example, Su et al. [7] screened 195 species of herbs, and 22 of them were found to be as effective as α-tocopherol, including 8 species that were more active than BHA. Some of the abovementioned herbs have been used for thousands of years in China (e.g., *Myristica fragrans*, *Poria cocos*, *Prinsepia uniflora*, etc.). Likewise, extracts of aromatic plants of Greek origin (such as *Taraxacum officinale*, *Crocus sativus*, *Asperulla odorata*, *Melissa officinalis*, *Origanum vulgare*, *Origanum dictamnus*, *Salvia officinalis* and *Hyssopus officinalis*) were examined as potential sources of phenolic compounds [8,9]. Despite the published reports on the topic, there are still species, native to Greece, that have not been explored, and may hold great promise. Although tocopherols are the most popular natural antioxidants in the food industry, it is well known that plants may contain a wide variety of free radical scavenging molecules, such as phenolic compounds (e.g., phenolic acids, flavonoids, quinones, coumarins, lignans, stilbenes, tannins), nitrogen compounds (alkaloids, amines, betalains), vitamins, terpenoids (including carotenoids), and some other endogenous metabolites, which present antioxidant activity [8]. Phenolic compounds are commonly found in both edible and nonedible plants, and they have been reported to have multiple biological effects, including antioxidant activity [10]. This activity is mainly due to their redox properties, which can play an important role in absorbing and neutralizing free radicals, quenching singlet and triplet oxygen, or decomposing peroxides [11]. The utility of these compounds as food lipid antioxidants is well known, having promoted studies of extracts from various plants containing them [12].

The recovery of phenols from plant tissues has so far been accomplished with various solvents including ethanol, methanol, and ethyl acetate. Methanol is an efficient solvent for the retrieval of antioxidant phenols from herbs [13,14]. In addition, Miliauskas et al. [15] examined the antioxidant activity of several acetone, ethyl acetate, and methanol extracts and showed that the methanolic ones were the most effective DPPH radical scavengers. Two conventional methods for determining the antioxidant activity of plants are the measurement of the phenolic content and radical scavenging activity. The Folin–Ciocalteu assay is the generally preferred method for measuring phenolics in plant-derived extracts that contain large amounts of polyphenols with antioxidant properties. Furthermore, it is important to select a stable and rapid method for the evaluation of antioxidant activity, because the determination of a large number of samples is time-consuming. Several methods have been developed to assay the free radical scavenging capacity and total antioxidant activity of plant extracts. The most common and reliable method involves the determination of the disappearance of free radicals such as 2,2-diphenyl-1-picrylhydrazyl radical (DPPH) using a spectrophotometer.

In addition, several chemical, instrumental, and sensory techniques are commonly used to monitor the oxidation in foods, predict their shelf stability, and evaluate their effectiveness as antioxidants in different lipid systems. Recently, several accelerated oxidation tests have been applied to examine the oxidative stability of edible oils and the ability of antioxidants to prolong their life [16]. The specificity and sensitivity of each method do not lead to a complete examination of all phenolic compounds in the examined extract. A combination of several tests could provide a more reliable assessment of its antioxidant activity [17]. Most methods are based on oxygen absorption and the formation of volatile oxidation products, e.g., the Rancimat method. However, other techniques, such as the Differential Scanning Calorimetry (DSC) method, have also been used for the investigation of the effects of flavonoids on the thermal auto-oxidation of palm oil and other vegetable oils [18].

The present study aimed to investigate possible new sources of natural antioxidants, which would be involved in the protection against diseases involving reactive oxygen species (ROS) and also be useful in food conservation. To this end, seventy methanolic extracts were prepared from fifty-seven Greek plant species (some of them not examined so far, to the best of our knowledge) and examined using the above-mentioned assays to obtain a better overview of their antioxidant capacity. The plants were collected from Crete, which is a Greek Island with unique flora, including interesting species and endemic plants. We aimed to study, highlight, and valorize these plant extracts as potential food additives. It is worth mentioning that the selected plant taxa, common and endemic, are good representatives of the Cretan flora.

## 2. Results and Discussion

### 2.1. Total Phenolics Content (TPC)

Since phenolics constitute one of the major groups of bioactive plant compounds that act as primary antioxidants or free radical terminators, it was reasonable to determine their total amount in the examined plant extracts. The total phenolics content (mg/g) of methanolic extracts was determined from a standard curve of gallic acid (*R*^2^ = 0.9934) and expressed as gallic acid equivalents (GAE), and it varied from 17.4 to 745.5 mg GAE/g of the extract (Table 1). The highest phenolic content was found in the extracts of *Cytinus* taxa (*C. hypocistis* subsp. *orientalis*, *C. ruber*, and *C. hypocistis* subsp. *hypocistis*), although high contents (>250 mg/g) were observed in the extracts of *Cistus monspeliensis*, *C. salviifolius*, *C. parviflorus*, *C. creticus* subsp. *creticus*, *C. creticus* subsp. *eriocephalus*, *Sarcopoterium spinosum*, *Staehelina petiolata*, and *Iris unguicularis* subsp. *cretensis*. In addition, significant amounts (>150 mg/g) of phenolic compounds were also contained the species *Origanum microphyllum*, *O. dictamnus*, *Daphne sericea*, *Rhamnus lycioides* subsp. *oleoides*, *Phlomis cretica*, *P. lanata*, *Sideritis syriaca* subsp. *syriaca*, *Berberis cretica* (fruits and aerial), *Ptilostemon chamepeuce*, *Salvia fruticosa*, *Anchusa cespitosa*, *Echinops spinosissimus* subsp. *spinosissimus*, *Verbascum spinosum*, *Cynoglossum columnae*, and *Parietaria cretica*.

Regarding the *Cytinus* taxa, there are only a few previous reports that examine these plants [19]. However, some phenolics have been identified, including phenolic acids (such as 5-*O*-caffeoylquinic acid), flavonoids (including flavones, apigenin derivatives, myricetin), and hydrolysable tannins (mainly gallotannins) [20]. The latter are of great importance because they can exhibit not only high antioxidant activity but also other bioactivities, such as antibacterial, anti-inflammatory, etc. [20]. Regarding the *Arum creticum* and *Arum idaeum* species, they were found to have almost the same content in polyphenols with *Arum dioscoridis* [21]. Additionally, the results obtained herein are in accordance with previous studies, which showed that the methanolic extracts of the above-mentioned extract are rich in polyphenols, such as tannins from *Cytinus* taxa [22], flavonoids, and catechin derivatives from *Cistus* species [23,24,25].

### 2.2. Evaluation of Antioxidant Activity

#### 2.2.1. DPPH Radical Scavenging Activity

The concentration of an antioxidant for decreasing the initial DPPH concentration by 50% (IC_50_) is a parameter widely used to measure antioxidant activity [26]. Between two samples, the one with the lower IC_50_ value exhibits the higher antioxidant activity. The scavenging activity of the plant extracts is shown in Table 1. It is noteworthy that all extracts that had a high phenolic content (>150 mg/g) showed a remarkable capacity to inhibit the DPPH radical (>80% at 200 μg/mL). The most effective DPPH radical scavengers (IC_50_ <50 μg/mL) were the extracts of *Cytinus* taxa (*C. hypocistis* subsp. *orientalis*, *C. ruber*, and *C. hypocistis* subsp. *hypocistis*), *Cistus monspeliensis*, *C. salviifolius*, *C. parviflorus*, *C. creticus* subsp. *creticus*, *C. creticus* subsp. *eriocephalus*, *Origanum microphyllum*, *Sarcopoterium spinosum*, *Cynoglossum columnae*, and *Daphne sericea*.

#### 2.2.2. Protection against Sunflower-Oil-Induced Oxidative Rancidity

The results represent a comparative study of the antioxidant activity of the sample extracts and known antioxidants (BHT and α-tocopherol) based on their protection factor. All sample extracts and antioxidants are presented at a concentration of 100 ppm. In most cases, a protection factor higher than 1 was recorded, as shown in Table 1. The sample of *Cytinus ruber* showed the highest protection factor (PF = 1.276) in the Rancimat method, which was similar to that of BHT (PF = 1.320). Additionally, the sample of *Berberis cretica* L. showed a significantly high protection factor (PF = 1.138), which was higher than that of α-tocopherol (PF = 1.090).

#### 2.2.3. Differential Scanning Calorimetry (DSC)

**Figure 1:**
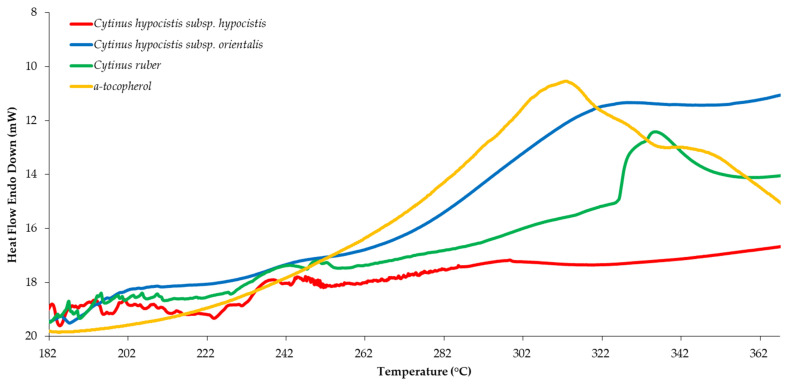
Thermal profile of plant extracts (family Rafflesiaceae) compared to α-tocopherol, as determined by the differential scanning calorimetry.

The thermal-oxidative decomposition of the pure extracts was studied using DSC. In comparison to the Rancimat method, DSC is concluded to be useful as a method employing milder conditions and a shorter time, which can be applied for the evaluation of the oxidative stability of samples containing volatile antioxidants and other lipid systems containing water [27]. An exothermic peak is observed in the range of 200 to 365 °C, related to the auto-oxidation process of the samples. Using the curves, the onset temperature (*T*_o_) at which the auto-oxidation process begins is determined [28]. *Cytinus* taxa (*C. hypocistis* subsp. *hypocistis*, *C. hypocistis* subsp. *orientalis*, and *C. ruber*) showed the highest oxidative stability in the DSC method. Owing to the results of the statistical analysis (*vide infra*), more emphasis was placed on the extracts from the Rafflesiaceae family. The effects of the thermal profile of pure extracts (family Rafflesiaceae) compared to α-tocopherol are shown in Figure 1. The onset temperature (*T*_o_) of the Rafflesiaceae family curves ranged from 300 to 335 °C and was similar to that of α-tocopherol (313 °C).

### 2.3. Statistics

A statistical analysis of the data presented in Table 1 was carried out in order to draw more conclusions. For the statistical analysis, only the plant extracts that exhibited significant antioxidant activity (≥50% scavenging of DPPH free radicals) were used.

**Figure 2:**
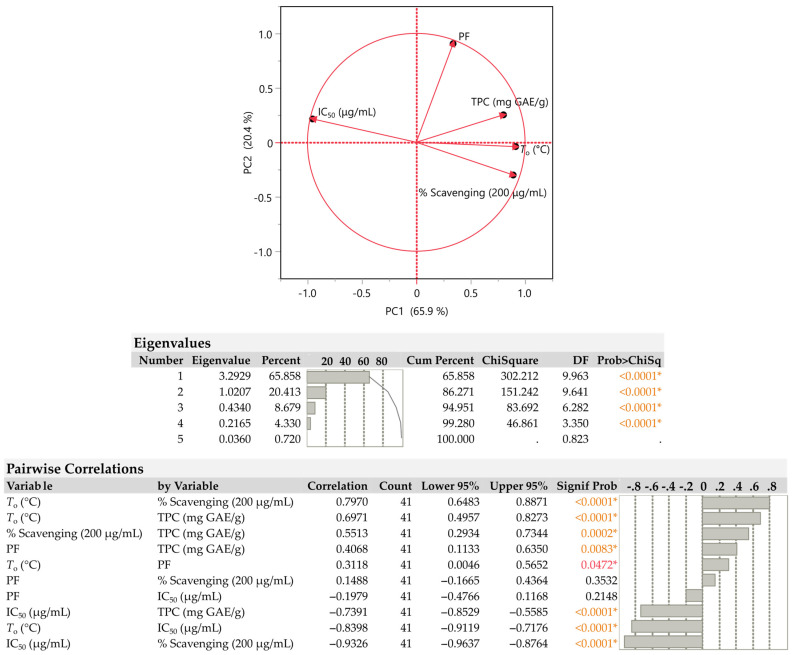
Plots for extracted plants using the principal component analysis (PCA). The axis scores for PC1 and PC2 were displayed. One of the five variables used in the PCA corresponds to each of the five separate bays, each of which has a different line assigned to it. Antioxidants and total phenolics content are examples of physicochemical properties. The physicochemical properties were estimated via pairwise correlation analysis. Statistically significant values are denoted by asterisks (*) and colored values.

In order to reduce the complexity of the multivariate data and obtain a better view of the results, a principal component analysis (PCA) was performed. As observed in Figure 2, the two main components that could account for 86.3% of the variation were chosen (Eigenvalues > 1), and this was considered to be a statistically significant parameter (*p* < 0.0001). PC1 demonstrated a positive association with TPC and antioxidant assays and a negative correlation with IC_50_, and it explained 65.9% of the variability. With a positive association between IC_50_, TPC, and PF and a negative correlation between *T*_o_ and the percentage of DPPH radicals reduced, PC2 can account for 20.4% of the variance in the data.

According to the PCA plot in Figure 2, TPC, *T*_o_, and DPPH all have nearly identical loading directions; however, PF has a different loading direction and clearly differs from the other variables in terms of IC_50_. As can be seen, TPC is more strongly, positively associated (>0.7) with the *T*_o_ parameter and is less strongly correlated (>0.4) with PF. Additionally, the highest correlation (0.797) was found between *T*_o_ and the % scavenging, which was found to be statistically significant (*p* < 0.0001). Furthermore, it is well known that the IC_50_ and % scavenging of DPPH radicals correlate negatively. A higher antioxidant activity is associated with lower IC_50_ concentrations. Thus, higher TPC concentrations are reflected in lower IC_50_ results.

**Figure 3:**
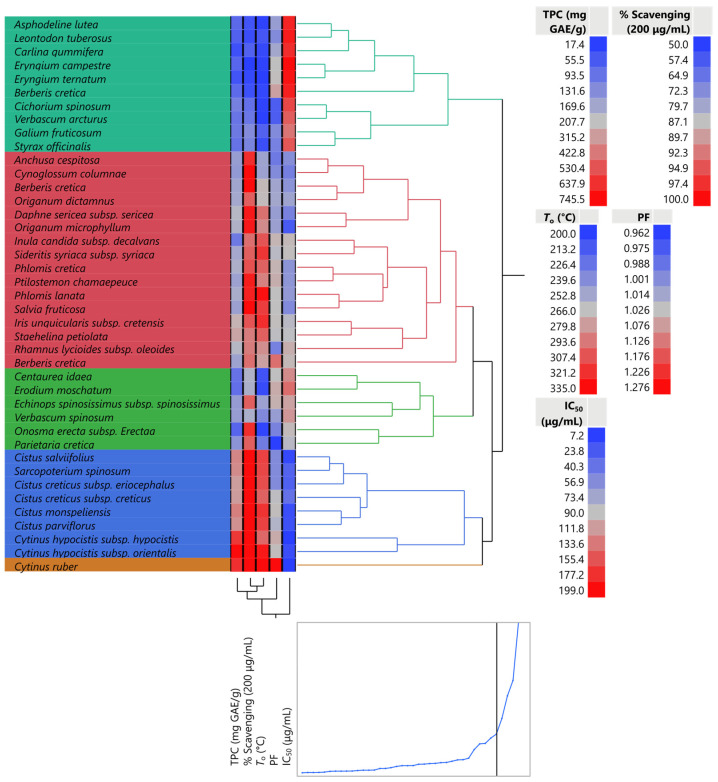
A hierarchical cluster analysis (using Ward’s method) of the plant extracts. The plot shows a dendrogram of hierarchical clustering.

The dendrogram that was created with the identification of the plant extracts that were considered to be the most comparable was the objective of the hierarchical cluster analysis. Ward’s method is the criterion applied in the hierarchical cluster analysis. *Cytinus ruber*, which offers a strong justification for its superiority compared to all other plant extracts, was clustered separately in Figure 3. Other members of the same family (Rafflesiaceae)—notably, *Cytinus hypocistis*—were likewise grouped separately, which may be viewed as strong support for its superiority to all other plant extracts.

**Figure 4:**
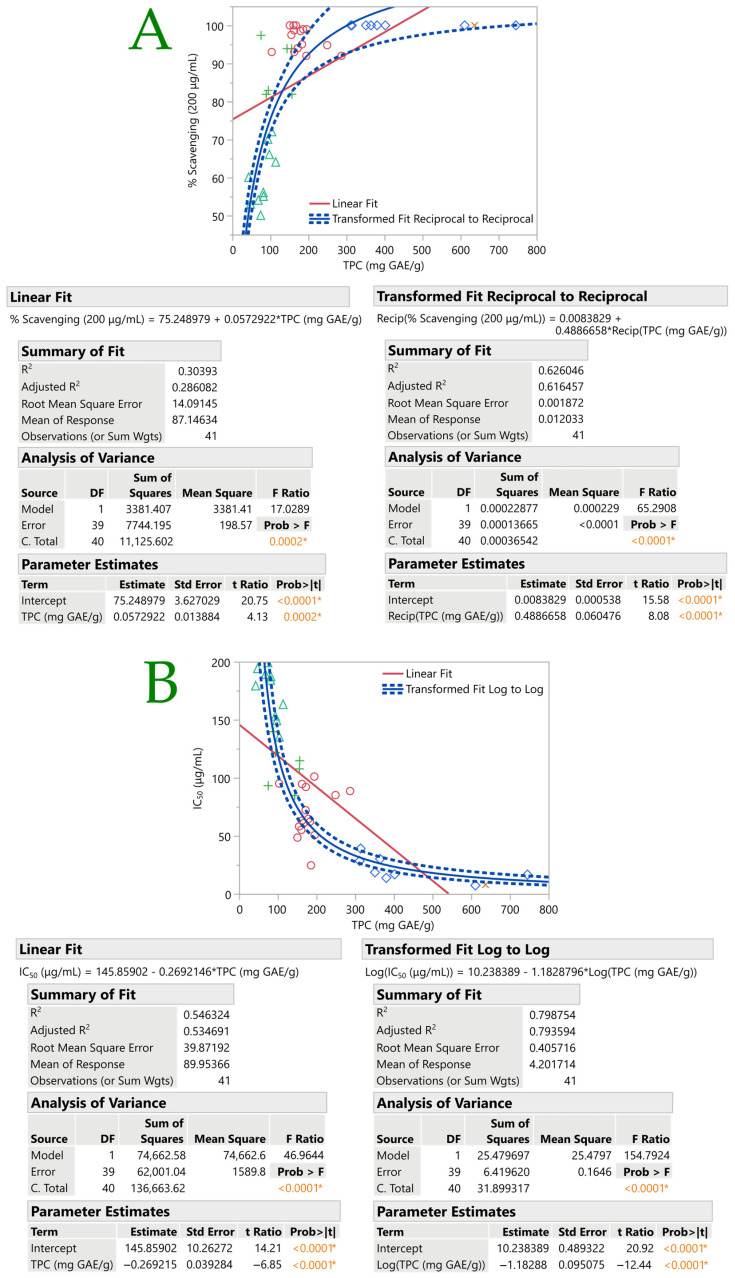

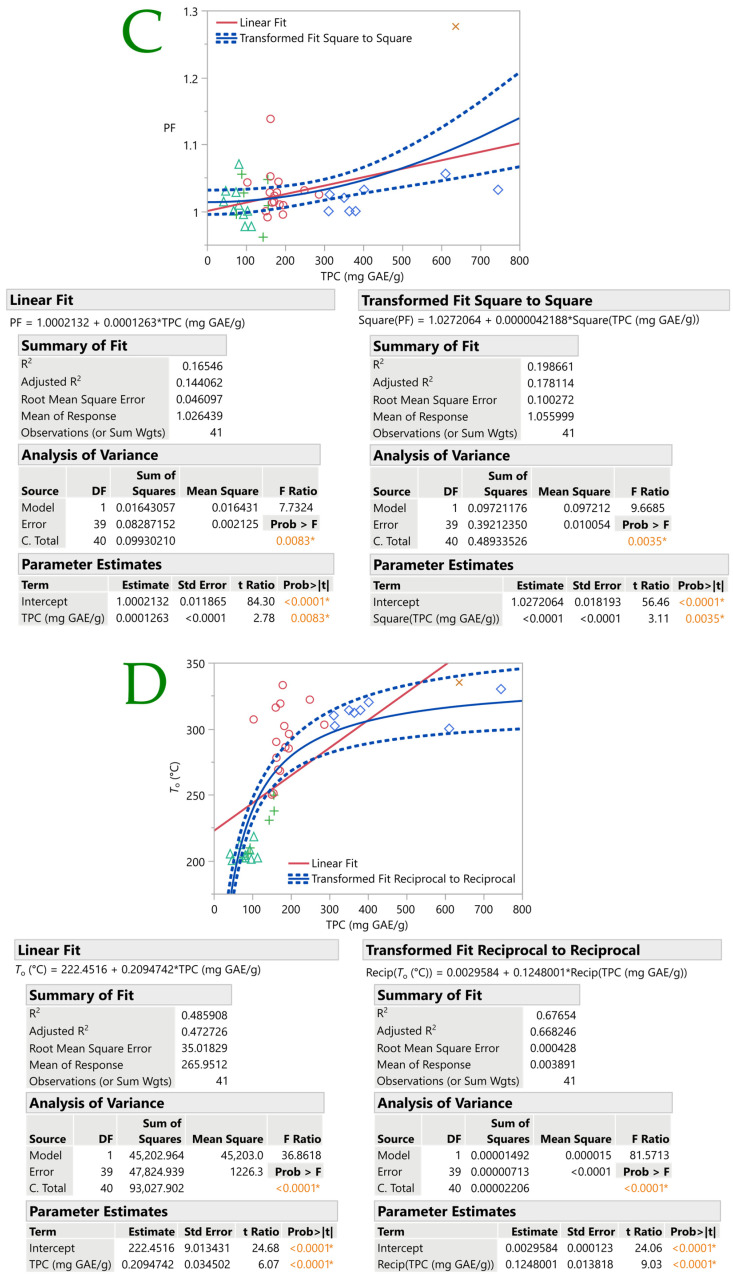
Antioxidant and total phenolics content (TPC) fit curves. Plots (**A**,**B**) show the relationship between the number of DPPH radicals that are scavenged and the TPC; plot (**C**) shows the relationship between the protection factor (PF) against oxidative rancidity and the TPC; and plot (**D**) shows the relationship between the onset temperature (*T*_o_) of the oxidation and the TPC. Row markers are used to distinguish the points in scatterplots. Statistically significant values are denoted by asterisks (*) and colored values.

Figure 4 shows the fit curves for antioxidant assays by TPC. In each plot, the linear fit and various statistics were displayed (i.e., equation, summary-of-fit, ANOVA, and parameter estimates). The linear fits, however, exhibited a low *R*^2^. Thus, curve fitting was carried out so as to have a better fit. Following that, the transformation fit had a higher *R*^2^ than the linear fit. Regarding the % DPPH scavenging in relation to the TPC, a reciprocal curve fit was found to be the most suitable, with an *R*^2^ value of 0.63. This was also the case for TPC and *T*_o_ (*R*^2^ = 0.68). A logarithmic plot curve was found to be the most suitable in explaining the relation between IC_50_ values and TPC (*R*^2^ = 0.80). Otherwise, a linear positive correlation between the total phenolic content and antioxidant activity was reported in the study of Skotti et al. [9].

## 3. Materials and Methods

### 3.1. Reagents

Methanol, dichloromethane, Folin–Ciocalteu reagent, sodium carbonate, 2,2-diphenyl-1-picrylhydrazyl hydrate (DPPH), butylated hydroxytoluene (BHT), α-tocopherol, and gallic acid were obtained from Sigma Aldrich (Steinheim, Germany).

### 3.2. Plant Material

The plant species and the parts used herein are presented in Table 2. The freshly collected plant parts were sorted out, dried in a room with active ventilation at ambient temperature, packed in bags, and stored at room temperature. All plants were collected in Crete, Greece, after 2017 and were identified by Dr. E. Kalpoutzakis. The voucher specimens were kept in the herbarium of the Laboratory of Pharmacognosy and Natural Products Chemistry, Department of Pharmacy, University of Athens, Greece. The specimen numbers and the places of the collection are also listed in Table 2. The plant families, genera, and species names are according to Dimopoulos et al. [29], except for the members of the genus *Cytinus* L., which are named in accordance with the Flora Europaea [30].

### 3.3. Preparation of the Plant Extracts

The pulverized plant materials (50 g) were defatted by maceration for 48 h with dichloromethane and subsequently extracted by maceration for 48 h with 0.5 L of methanol (analytical grade). The extraction step was repeated two more times. The three methanolic extracts were combined. Next, the organic solvent was removed by vacuum distillation. All residues were then stored in a dry place protected from light.

### 3.4. Determination of Total Phenolics in the Extracts

The concentration of total phenolic compounds in the MeOH extracts was determined spectrometrically using the Folin–Ciocalteu method [31], using gallic acid as a standard to prepare a calibration curve. A total of 1 mL of plant extract (10 g/L) was mixed with 5 mL of Folin–Ciocalteu reagent and 4 mL (75 g/L) of sodium carbonate, and after 1 h, the absorption of the reaction mixture was measured at 765 nm against a methanol blank, using a Shimadzu UV-1700 UV/vis spectrophotometer (Tokyo, Japan). The results were expressed as milligrams of gallic acid equivalent (GAE) per gram of extract, based on the reference gallic acid calibration curve (at a linearity range of 1–10 μg/mL, with the equation y = 0.0834x + 0.0925 and R^2^ = 0.9967) generated for this study. All determinations were performed in triplicate.

### 3.5. Evaluation of Antioxidant Activity

#### 3.5.1. DPPH Radical Scavenging Assay

The radical scavenging activity of the plant extracts against stable DPPH was determined spectrometrically according to a previously reported procedure [32]. Briefly, 100 μL of the sample solution (200 mg/L), diluted in dimethylsulfoxide, was added to 1.9 mL of a 315 μM DPPH solution (in ethanol) and allowed to react for 30 min at 37 °C. A blank sample was prepared by adding 100 μL of dimethylsulfoxide in the DPPH solution. Then, the absorbance was measured at 515 nm, and the % scavenging was calculated using the following equation:

(1) $$\%\;\mathrm{Scavenging}=\left(\frac{A_{0}-\;A}{A_{0}}\right)\;\times \;100$$

where *A*_0_ and *A* are the absorbances of the blank solution and the sample, respectively.

The IC_50_ values correspond to the amount of each sample required to scavenge 50% of the DPPH free radicals. They were calculated from regression lines, where the abscissa represents the sample concentration, and the ordinate is the average percent reduction of the DPPH radical. Each IC_50_ value corresponds to an average of three separate tests. Plant extracts that achieved lower than 50% scavenging of DPPH radicals were not further examined.

#### 3.5.2. Protection against the Oxidative Rancidity of Sunflower Oil

The method used was adapted from Lalas and Tsaknis [33]. Two and a half grams of sunflower oil and an antioxidant (plant extract, BHT, or α-tocopherol, in various concentrations) were accurately weighed into the reaction vessel of a Rancimat 679 (Metrohm LTD, Herisau, CH 9101, Switzerland). At the same time, in another vessel, pure sunflower oil (iodine value: 115 g I/100 g) (Elais S.A., Athens, Greece) was added (without antioxidants) to be considered as a control sample. A total of 1 mL of the appropriate solvent (methanol or dichloromethane) was added in order to dissolve the antioxidant and mixed well. The conditions were set at a temperature of 90 °C and an airflow of 15 L/h. The protection factor (PF) was calculated as follows: PF = (induction period with antioxidant)/(induction period without antioxidant). A protection factor greater than 1 indicates the inhibition of lipid oxidation. The higher the value, the better the antioxidant activity [33].

#### 3.5.3. Differential Scanning Calorimetry (DSC)

The antioxidant action of extracts was estimated using the DSC method with a Perkin Elmer DSC-6 calorimeter (Perkin Elmer Corp., Norwalk, CT, USA). Oxidative stability was determined using the method of Tan and Che Man [34]. A total of 4 mg of the sample extracts (or α-tocopherol for comparison) was placed in DSC aluminum pans closed with lids perforated by a hole (internal diameter: 1 mm) in the center in order to allow the sample to be in contact with the oxygen stream. The purge gas foaming the reaction atmosphere was oxygen. The starting temperature of oxidation was determined as the onset temperature of the oxidation peak. The temperature program was: heat from 30 °C to 180 °C (at a rate of 100 °C/min), hold for 1 min at 180 °C, and, finally, heat from 180 °C to 390 °C (at a rate of 10 °C/min).

### 3.6. Statistics

Principal component analysis (PCA), hierarchical cluster analysis, and statistical analysis were all carried out using the JMP^®^ Pro 16 (SAS, Cary, NC, USA) software. Each plant extract was subjected to three separate analyses, with three replicates of each determination described above.

## 4. Conclusions

During the screening of fifty-seven plants in this work, Cytinus taxa (*C. hypocistis* subsp. hypocistis, *C. hypocistis* subsp. orientalis, and *C. ruber*), Cistus species (*C. creticus* subsp. creticus, *C. creticus* subsp. eriocephalus, *C. monspeliensis*, *C. parviflorus*, and *C. salviifolius*), and Sarcopoterium spinosum were found to be the most promising ones. All these extracts showed a high phenolic concentration and significant free radical scavenging activity. Since the reports for the TPC and antioxidant activity of most of the examined plant species are scanty and sparse, the results of this study can be used as a benchmark for future studies on the same plant species. Moreover, plant species that were overlooked or not thoroughly examined were highlighted as potential candidates so that they can be further studied and used for industrial purposes.

## Figures and Tables

**Table 1:** Total phenolics content (TPC), DPPH radical scavenging activity, protection factor (PF), and onset temperature (*T*_o_) of curves of the plant extracts.

Plant Species	Plant Part	TPC (mg GAE/g) ± SD	DPPH		PF	*T*_o_ (°C)
			% Scavenging (200 μg/mL)	IC_50_ (μg/mL) ± SD		
*Anchusa cespitosa*	whole	155.2 ± 3.6 *	97.5	58 ± 2	0.991	251
*Aristolochia cretica*	aerial	53.0 ± 2.4	<50	– **	–	–
	radix	50.7 ± 1.9	<50	–	–	–
*Arum creticum*	aerial	63.2 ± 3.0	<50	–	–	–
	rhizome	58.8 ± 2.4	<50	–	–	–
*Arum idaeum*	aerial	72.0 ± 3.9	<50	–	–	–
	rhizome	63.3 ± 3.3	<50	–	–	–
*Asphodeline lutea*	rhizome	67.2 ± 1.5	<50	–	–	–
	aerial	81.4 ± 3.7	56	184 ± 4	1.009	202
*Astragalus angustifolius* subsp. *echinoides*	aerial	74.6 ± 3.4	<50	–	–	–
	rhizome	65.2 ± 2.7	<50	–	–	–
*Astragalus creticus* subsp. *creticus*	aerial	74.4 ± 3.8	<50	–	–	–
	rhizome	17.4 ± 0.9	<50	–	–	–
*Carlina gummifera*	aerial	42.8 ± 2.2	60	179 ± 5	1.014	205
	rhizome	44.2 ± 1.9	<50	–	–	–
*Bellis longifolia*	whole	67.4 ± 1.5	<50	–	–	–
*Berberis cretica*	fruit	167.4 ± 7.9	100	61 ± 2	1.013	269
	radix	82.0 ± 2.6	55	187 ± 4	1.070	204
	aerial	162.8 ± 3.9	93	94.5 ± 3.3	1.138	278
*Bryonia cretica*	aerial	73.5 ± 1.6	<50	–	–	–
*Campanula tubulosa*	whole	86.2 ± 4.4	<50	–	–	–
*Centaurea idaea*	aerial	93.2 ± 3.4	83	122 ± 3	1.028	210
*Centaurea raphanina* subsp. *raphanina*	aerial	60.8 ± 2.4	<50	–	–	–
*Cichorium spinosum*	aerial	113.9 ± 4.2	64	163 ± 4	0.977	202
*Cistus salviifolius*	aerial	380.6 ± 19.0	100	13.7 ± 0.4	1.000	314
*Cistus creticus* subsp. *creticus*	aerial	314.2 ± 14.5	100	39 ± 1.4	1.025	302
	resin	83.0 ± 1.8	<50	–	–	–
*Cistus creticus* subsp. *eriocephalus*	aerial	311.6 ± 16.8	100	28.3 ± 1.0	1.000	310
*Cistus monspeliensis*	aerial	402.2 ± 13.7	100	16.7 ± 0.5	1.032	320
*Cistus parviflorus*	aerial	351.2 ± 19.3	100	18.5 ± 0.6	1.020	314
*Cynoglossum columnae*	aerial	150.9 ± 7.5	100	48.4 ± 1.6	1.000	250
*Cytinus hypocistis* subsp. *hypocistis*	whole	611 ± 15.3	100	7.2 ± 0.2	1.056	300
*Cytinus hypocistis* subsp. *orientalis*	whole	745.5 ± 32.8	100	16.5 ± 0.3	1.032	330
*Cytinus ruber*	whole	637 ± 35.0	100	7.8 ± 0.3	1.276	335
*Daphne sericea* subsp. *sericea*	aerial	195.3 ± 7.8	99	50.5 ± 1.1	1.009	296
*Echinops spinosissimus* subsp. *spinosissimus*	aerial	154.7 ± 4.0	94	108 ± 2	1.048	250
	radix	71.6 ± 2.4	<50	–	–	–
*Erodium moschatum*	aerial	88.0 ± 4.8	82	140 ± 3	1.056	206
*Eryngium amorginum*	aerial	40.0 ± 1.2	<50	–	–	–
*Eryngium campestre*	aerial	74.6 ± 3.8	50	199 ± 4	1.028	202
*Eryngium creticum*	aerial	67.2 ± 1.7	<50	–	–	–
*Eryngium maritimum*	aerial	43.9 ± 1.6	<50	–	–	–
*Eryngium ternatum*	aerial	48.0 ± 2.4	53	194 ± 4	1.030	200
*Galium fruticosum*	aerial	104.0 ± 4.2	72	135 ± 5	1.000	218
*Helminthotheca echioides*	aerial	47.4 ± 1.0	<50	–	–	–
*Inula candida* subsp. *decalvans*	aerial	103.5 ± 5.2	93	95 ± 3	1.043	307
*Iris unguicularis* subsp. *cretensis*	rhizome	249.4 ± 6.2	94.8	85 ± 2	1.031	322
*Leontodon tuberosus*	whole	68.1 ± 2.2	54	189 ± 4	1.000	212
*Alyssoides cretica*	aerial	58.9 ± 2.6	<50	–	–	–
*Nepeta melissifolia*	aerial	40.1 ± 1.2	<50	–	–	–
*Onosma erecta* subsp. *Erectaa*	aerial	74.1 ± 2.0	97.5	93.5 ± 2.6	0.996	203
*Origanum dictamnus*	aerial	172 ± 8.6	94	72 ± 2	1.014	268
*Origanum microphyllum*	aerial	186 ± 8.4	99	24.5 ± 0.9	1.010	286
*Parietaria cretica*	aerial	142.6 ± 3.0	94	85 ± 3	0.962	231
*Petromarula pinnata*	aerial	51.4 ± 1.7	<50	–	–	–
*Phlomis cretica*	aerial	183.1 ± 9.9	95	62 ± 2	1.044	302
*Phlomis lanata*	aerial	179.1 ± 4.7	98.5	64.5 ± 2.4	1.028	333
*Ptilostemon chamaepeuce*	aerial	162.4 ± 7.1	98.7	63 ± 2	1.052	290
*Rhamnus lycioides* subsp. *oleoides*	aerial	194.5 ± 9.7	92	101 ± 3	0.995	285
*Salvia fruticosa*	aerial	160.9 ± 3.9	100	55 ± 1	1.028	316
*Sarcopoterium spinosum*	aerial	364.6 ± 13.5	100	30 ± 0.6	1.000	312
*Sideritis syriaca* subsp. *syriaca*	aerial	172.8 ± 4.7	94	92 ± 2	1.023	319
*Stachys spinosa*	aerial	67.4 ± 1.9	<50	–	–	–
*Staehelina petiolata*	aerial	287.0 ± 14.4	92	88.5 ± 1.9	1.025	303
*Styrax officinalis*	stems	93.6 ± 5.1	70	153 ± 2.6	0.995	208
	flowers	48.2 ± 1.2	<50	–	–	–
*Tordylium apulum*	aerial	84.8 ± 3.6	<50	–	–	–
	rosette	74.9 ± 2.6	<50	–	–	–
*Verbascum arcturus*	aerial	97.4 ± 3.5	66	149 ± 4	0.977	201
*Verbascum spinosum*	aerial	155.5 ± 4.5	82	115 ± 4	1.009	238
Gallic acid				4.8 ± 0.2		
α-tocopherol					1.090	313

* TPC and IC_50_ results are expressed as the mean ± SD (n = 3); ** Not calculated.

**Table 2:** Plants of the Cretan flora that were investigated.

Name	Family	Plant Part	Voucher	Yield of Extract g/50 g of Plant Material	Origin
*Anchusa cespitosa* Lam. ^a^	Boraginaceae	Whole	KL064	4.3	West Crete
*Aristolochia cretica* Lam. ^a^	Aristolochiaceae	Rhizome	KL001R	6.6	East Crete
		Aerial	KL001Y	5.9	
*Arum creticum* Boiss. & Heldr.	Araceae	Bulbs	KL002R	8.1	Central Crete
		Aerial	KL002Y	6.2	
*Arum idaeum* Coustur. & Gand. ^a^	Araceae	Aerial	KL003Y	5.9	West Crete
		Bulbs	KL003R	7.9	
*Asphodeline lutea* (L.) Rchb.	Asphodelaceae	Aerial	KL065Y	5.7	Central Crete
		Rhizome	KL065R	7.8	
*Astragalus angustifolius* subsp. *echinoides* (L’Hér.) Brullo & al. ^a^	Fabaceae	Aerial	KL067Y	5.8	Central Crete
		Rhizome	KL067R	7.3	
*Astragalus creticus* Lam. subsp. *creticus* ^a^	Fabaceae	Aerial	KL004Y	5.9	Central Crete
		Rhizome	KL004R	5.7	
*Carlina gummifera* (L.) Less.	Asteraceae	Aerial	KL005Y	6.1	Central Crete
		Rhizome	KL005R	7.8	
*Bellis longifolia* Boiss. & Heldr. in Boiss. ^a^	Asteraceae	Whole	KL068	6.9	West Crete
*Berberis cretica* L.	Berberidaceae	Radix	KL006R	7.6	Central Crete
		Fruits	KL006F	5.5	
		Aerial	KL006Y	6.7	
*Bryonia cretica* L.	Cucurbitaceae	Aerial	KL007Y	6.2	East Crete
*Campanula tubulosa* Lam. ^a^	Campanulaceae	Whole	KL008	6.7	Central Crete
*Centaurea idaea* Boiss. & Heldr. ^a^	Asteraceae	Whole	KL009	6.4	Central Crete
*Centaurea raphanina* Sm. subsp. *raphanina* ^a^	Asreraceae	Whole	KL010	7.1	Central Crete
*Cichorium spinosum* L.	Asreraceae	Whole	KL011	6.1	Central Crete
*Cistus salviifolius* L.	Cistaceae	Aerial	KL059	5.6	Central Crete
*Cistus creticus* L. subsp. *creticus*	Cistaceae	Aerial	KL057	5.8	Central Crete
		Resin	KL057R	6.6	
*Cistus creticus* subsp. *eriocephalus* (Viv.) Greuter & Burdet	Cistaceae	Aerial	KL058	6.3	Central Crete
*Cistus monspeliensis* L.	Cistaceae	Aerial	KL060	6.2	East Crete
*Cistus parviflorus* Lam.	Cistaceae	Aerial	KL012	5.9	Central Crete
*Cynoglossum columnae* Ten.	Boraginaceae	Aerial	KL013b	7.1	Central Crete
*Cytinus hypocistis* (L.) L. subsp*. hypocistis*	Rafflesiaceae	Whole	KL014	19.1	Central Crete
*Cytinus hypocistis* subsp. *orientalis* Wettst.	Rafflesiaceae	Whole	KL015	15.2	West Crete
*Cytinus ruber* (Fourr.) Willd.	Rafflesiaceae	Whole	KL016	16.5	Central Crete
*Daphne sericea* Vahl subsp. *sericea*	Thymelaeacea	Aerial	KL070	6.2	West Crete
*Echinops spinosissimus* Turra subsp. *spinosissimus*	Asteraceae	Aerial	KL018Y	4.1	Central Crete
		Radix	KL018R	3.9	
*Erodium moschatum* (L.) L’Hér.	Geraniaceae	Aerial	KL019	6,2	Central Crete
*Eryngium amorginum* Rech. fil. ^a^	Apiaceae	Aerial	KL100	4.7	East Crete
*Eryngium campestre* L.	Apiaceae	Aerial	KL107	4.5	Central Crete
*Eryngium creticum* Lam.	Apiaceae	Aerial	KL020	4.7	West Crete
*Eryngium maritimum* L.	Apiaceae	Aerial	KL021	4.1	West Crete
*Eryngium ternatum* Poir. ^a^	Apiaceae	Aerial	KL022	4.1	West Crete
*Galium fruticosum* Willd.	Rubiaceae	Aerial	KL074	5.7	West Crete
*Helminthotheca echioides* (L.) Holub	Asteraceae	Aerial	KL031	5.3	Central Crete
*Inula candida* subsp. *decalvans* (Halácsy) Tutin ^a^	Asteraceae	Aerial	KL071	6.1	East Crete
*Iris unguicularis* Poir. subsp. *cretensis* (Janka) A.P. Davis & Jury ^a^	Iridaceae	Rhizome	KL024	6.6	Central Crete
*Leontodon tuberosus* L.	Asteraceae	Whole	KL038	6.8	Central Crete
*Alyssoides cretica* (L.) Medik. ^a^	Brassicaceae	Aerial	KL072	5.7	East Crete
*Nepeta melissifolia* Lam. ^a^	Lamiaceae	Aerial	KL103	6.3	East Crete
*Onosma erecta* Sm. subsp*. erecta* ^a^	Boraginaceae	Aerial	KL025	4.1	West Crete
*Origanum dictamnus* L. ^a^	Lamiaceae	Aerial	KL026	6.1	Central Crete
*Origanum microphyllum* (Benth.) Vogel ^a^	Lamiaceae	Aerial	KL078	5.7	East Crete
*Parietaria cretica* L.	Urticaceae	Aerial	KL027	4.2	West Crete
*Petromarula pinnata* (L) A. DC. ^a^	Campanulaceae	Aerial	KL028	4.5	Central Crete
*Phlomis cretica* C. Presl ^a^	Lamiaceae	Aerial	KL029	4.9	Central Crete
*Phlomis lanata* Willd. ^a^	Lamiaceae	Aerial	KL030	5.2	Central Crete
*Ptilostemon chamaepeuce* (L.) Less.	Asteraceae	Aerial	NEK009	4.7	West Crete
*Rhamnus lycioides* subsp. *oleoides* (L.) Jahand. & Maire	Rhamnaceae	Aerial	KL032	5.8	Central Crete
*Salvia fruticosa* Mill.	Lamiaceae	Aerial	KL053B	5.1	Central Crete
*Sarcopoterium spinosum* (L.) Spach	Rosaceae	Aerial	KL033	5.7	Central Crete
*Sideritis syriaca* L. subsp. *syriaca* ^a^	Lamiaceae	Flowering stems	KL035	5.3	Central Crete
*Stachys spinosa* L. ^a^	Lamiaceae	Aerial	KL036	5.7	Central Crete
*Staehelina petiolata* (L.) Hilliard & B.L. Burtt ^a^	Asteraceae	Aerial	KL073	6.1	Central Crete
*Styrax officinalis* L.	Styracaceae	Stems	KL037K	6.3	Central Crete
		Flowers	KL037F	3.5	
*Tordylium apulum* L.	Apiaceae	Rosette	KL039R	4.3	Central Crete
		Aerial	KL039	3.9	
*Verbascum arcturus* L. ^a^	Scrophulariaceae	Aerial (annual)	KL040Y	5.5	West Crete
*Verbascum spinosum* L. ^a^	Scrophulariaceae	Aerial	KL048	5.2	West Crete

^a^: Endemic plants of Greece.

## Data Availability

Not applicable.
